# Evaluation of Tropicamide–Phenylephrine Mydriatic Eye Drop Instillation on Choroidal Thickness

**DOI:** 10.3390/jcm12196355

**Published:** 2023-10-04

**Authors:** Marco Gioia, Maddalena De Bernardo, Sergio Pagliarulo, Ferdinando Cione, Francesco Ferdinando Mottola, Aniello La Marca, Ilaria De Pascale, Giovanni Albano, Nicola Rosa

**Affiliations:** Eye Unit, Department of Medicine Surgery and Dentistry, “Scuola Medica Salernitana”, University of Salerno, 84081 Baronissi, Italy; mgioia@unisa.it (M.G.); mdebernardo@unisa.it (M.D.B.); nrosa@unisa.it (N.R.)

**Keywords:** choroidal thickness, tropicamide, phenylephrine, choroid, OCT, EDI-OCT

## Abstract

The purpose of this study is to evaluate choroidal thickness (ChT) at the subfoveal and peripheral level after the instillation of 0.5% tropicamide + 10% phenylephrine 9 hydrochloride eye drops by using OCT scans in enhanced depth image (EDI) mode. In total, 53 patients (30 males and 23 females) were involved, and the mean age was 25.62 ± 2.41 (age range: 23–36). The dominant eye was treated with tropicamide + phenylephrine (Visumidriatic Fenil 100 mg/mL + 5 mg/mL, Visufarma) while the nondominant eye was used as the control. An OCT analysis was performed on both eyes before and 30 min after the instillation of a drop of mydriatic in the dominant eye. The ChT was measured by using the OCT software measurement tool (Spectralis; Heidelberg Engineering; Heidelberg, Germany, version 6.0). The results showed a statistically significant ChT decrease (*p* = 0.009) in the temporal sector after the treatment with tropicamide + phenylephrine. In the subfoveal and nasal sectors, no statistically significant ChT changes were detected (*p* = 0.94; *p* = 0.85) following the administration of the mydriatic eye drops. The ChT thinning in the temporal sector following the instillation of the tropicamide + phenylephrine eye drops suggests that in the case of ChT studies, mydriatic administration should be avoided.

## 1. Introduction

The choroid is a thin (60–300 micron), highly vascularized pigmented layer that covers the posterior 5/6 of the eye, extending anteriorly to the ora serrata, where it continues with the ciliary body. Together with the ciliary body and the iris, it forms the uvea, the vascular layer of the eye [1,2]. Its main function is to supply oxygen and nutrients to the retina [1,2].

In cross-sections, four layers can be identified, namely [3,4,5] Bruch’s membrane, choriocapillaris, the vascular stroma and the suprachoroid.

The choroid is richly innervated by parasympathetic and sympathetic nerve fibers.

Parasympathetic activity has been shown to be responsible for vasodilation and increased choroidal blood flow, while sympathetic input induces vasoconstriction and decreased choroidal blood flow [4,6].

Muscarinic cholinergic antagonists and sympathetic mimetic agents are commonly used in the form of eye drops in ophthalmic practice for fundus examination, cycloplegia and surgery. Among them are:

- Phenylephrine [7], a selective sympathetic mimetic receptor agonist 1, available in different formulations (2.5% and 10%) as mydriatic eye drops.
- Tropicamide [7], a muscarinic antagonist available in various formulations (0.5% and 1%) which is used in the ophthalmic field for cycloplegia and mydriasis.

The correlation between the use of mydriatics and choroidal thickness (ChT) has been evaluated in several papers with rather conflicting results [8,9,10,11,12,13]. Some studies revealed no significant ChT changes [9,10,11,12,13] while others have shown significant choroidal thinning following the instillation of mydriatic eye drops [8,10,11,12].

In the last years, choroidal imaging received a great improvement thanks to a new standard technique, namely enhanced depth imaging–optical coherence tomography (EDI-OCT), which allows one to clearly visualize the vascular tissue underneath the retina [14].

The aim of this study is to evaluate the influence of tropicamide + phenylephrine by measuring the ChT at the subfoveal, 1500 micron nasal and temporal levels by using OCT scans in enhanced depth image (EDI) mode.

## 2. Materials and Methods

### 2.1. Patient Selection

In this study, healthy subjects evaluated at the Eye Unit, Department of Medicine, Surgery and Dentistry, “Scuola Medica Salernitana”, University of Salerno, Salerno, Italy, were recruited for the period from January 2020 to November 2021. Subjects with systemic and ocular pathologies that could have influenced the measurements, such as diabetes, hypertension, smoking, carotid artery stenosis, Parkinson’s disease, Alzheimer’s disease or cognitive impairment and ocular conditions such as pseudoexfoliation syndrome and amblyopia were excluded from this study [15,16,17,18,19,20,21,22,23]; additionally, patients who had undergone ocular surgery, patients under 18 or over 70 and pregnant patients were not included in this work. All the participants were thoroughly informed about the purpose of this study, and written informed consent was obtained from each of them.

### 2.2. Clinical and Instrumental Examinations

All the patients underwent a complete ophthalmological evaluation, both before and after the tropicamide + phenylephrine drop (Visumidriatic Fenil 100 mg/mL + 5 mg/mL, Visufarma) administration. The evaluation included an axial length (AL) measurement with IOLMaster (Carl Zeiss Meditec AG, Jena, Germany, version 5.4.0006), taking into account the recently suggested changes [24] in corneal thickness with Pentacam HR and Nidek CEM-530 via a spectral domain (SD) OCT evaluation (Spectralis; Heidelberg Engineering; Heidelberg, Germany, version 6.0) with EDI mode. Clinical refraction was not assumed as a primary parameter of this study because it could be influenced not only by the AL but also by the mean keratometry, lens opacity or accommodative state. For this reason, the AL was assumed as the main parameter to stratify the subjects of this study.

The eye to be treated was chosen based on ocular dominance [25,26] derived from a series of tests performed by two operators; the fellow eye was utilized as the control. The tests performed were:

- The hole-in-the-card test: The subject holds, with two hands and extended arms, a sheet with a central hole of 3 cm in diameter. The task of the examinee is to look at a target through the hole. The eye looking at the target is the dominant one [27].
- The convergence test: An object, a pen, is slowly brought close to the subject in the median plane at nose level, inviting him to fixate on it. The eye that loses fixation is the nondominant one [28]. The distance of the pen to the nose that determines the loss of fixation of the nondominant eye was variable depending on the subject.
- The sighting test: The subject is asked to bring his hands together with palms outward and arms extended so that a small space remains between the thumbs and fingers. The examinee is asked to look at a target through this space. The eye visible to the examiner through the space between the fingers is the dominant eye [29].

The dominant eye was determined when the results of at least two out of the three tests were in agreement, whereas the fellow nondominant eye was used as a control. Vergence, accommodative or motility dysfunctions were ruled out in order to apply these tests. All the subjects had a corrected visual acuity of 0.0 logMAR (1.0 in decimals) in both eyes. None of the examined subjects had any kind of strabismus or other sources of monovision.

### 2.3. OCT Analysis

An OCT analysis was performed on both eyes before and at least 30 min after a drop of the tropicamide + phenylephrine instillation in the dominant eye. The patients were instructed not to smoke [30], consume coffee [31] or exercise [32] 30 min prior to the ophthalmic evaluation and OCT analysis as this has also been suggested to influence the subfoveal choroidal thickness. All the evaluations were performed on the same time frame each day, in the early afternoon, as diurnal variation could also affect the choroidal thickness [33].

A horizontal 30° linear OCT B scan passing through the fovea was acquired by an expert examiner not aware of the patients’ distribution with a scanning angle of 308 and with 100 frames per B scan, with and without an EDI approach.

Only EDI-OCT [14,34] images with a high signal-to-noise ratio (minimum of 20 dB) that permitted the sufficient choroidal visualization of the choroid were included. All the examinations were performed between 2:00 p.m. and 3:00 p.m., reducing the bias related to the diurnal variations in choroidal thickness.

All the measurements were taken with the built-in software of the device (Heidelberg Eye Explorer HEYEX, version 5.3; Heidelberg Engineering).

All the collected and analyzed imaging data were reviewed by an expert in OCT evaluation (MDB).

The ChT was measured by using the OCT software measurement tool (Spectralis; Heidelberg Engineering; Heidelberg, Germany, version 6.0): a line was drawn at the fovea running from the retinal pigment epithelium (EPR) to the chorioscleral border in the EDI scans, allowing for an optimal choroidal view. Starting from the fovea, 1500 micron lines were drawn nasally and temporally to allow for the assessment of the nasal and temporal ChT relative to the fovea.

### 2.4. Statistical Analysis

Data collection was performed with Microsoft Excel, and statistical analyses were performed with IBM SPSS Statistics Version 26 (International Business Machine Corporation, Armonk, NY, USA). A Kolmogorov–Smirnov test was performed to determine the normality of the data distribution (*p* > 0.05). In addition, the mean, standard deviation, minimum and maximum were calculated for each group and for each parameter under study. Pre- and postadministration measurements of tropicamide + phenylephrine were compared by using paired Student’s *t*-tests. Correlations between the choroidal thickness before and after the eye drop instillation both in the dominant and nondominant eyes were evaluated by Pearson’s correlation coefficient (r). *p*-values of less than 0.05 were considered statistically significant.

The required sample size was obtained with G*Power software (Version 3.1.9.7, Faul, Erdfelder, Lang and Buchner). The sample size was calculated with a paired *t*-test as follows: α error = 0.05, 1 − β error = 0.95 and effect size = 0.46 were set, and the noncentral parameter δ = 3.35, critical t = 1.67, Df = 52, total simple size = 53 and actual power = 0.951 were obtained.

## 3. Results

A total of 53 patients (30 males and 23 females) were included in this study; the mean age was 25.62 ± 2.41 (age range: 23–36) and the AL was 24.52 ± 1.31 mm (from 20.82 to 29.07 mm).

**Figure 1:**
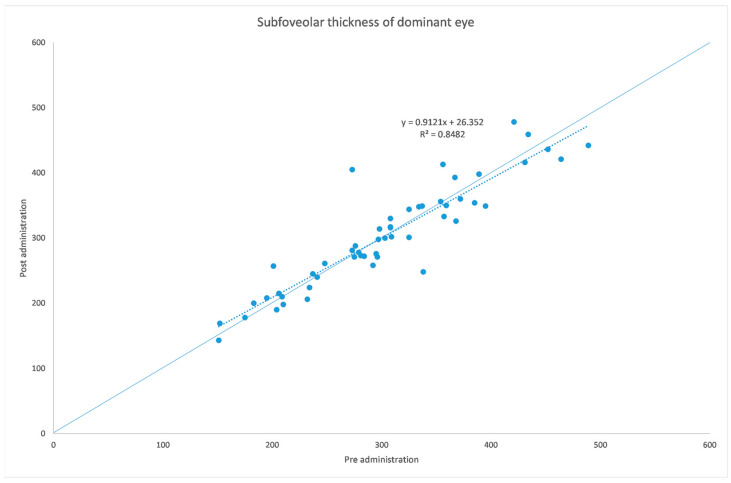
Subfoveal ChT before and after administration of tropicamide + phenylephrine in the treated eye.

**Figure 2:**
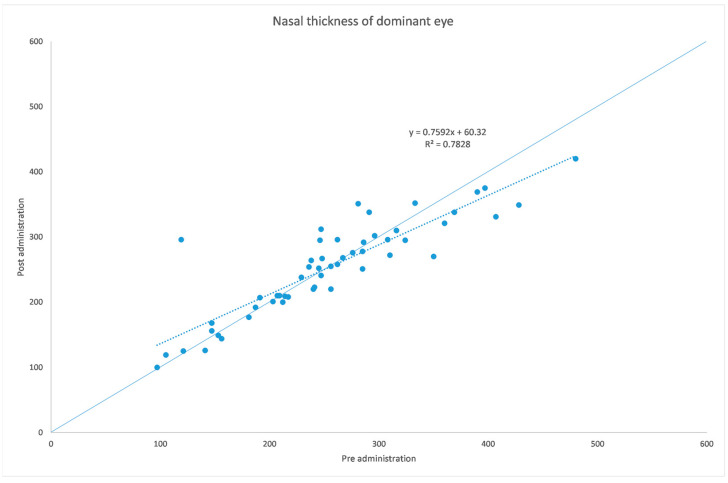
Choroidal thickness at nasal level before and after administration of tropicamide + phenylephrine in the treated eye.

**Figure 3:**
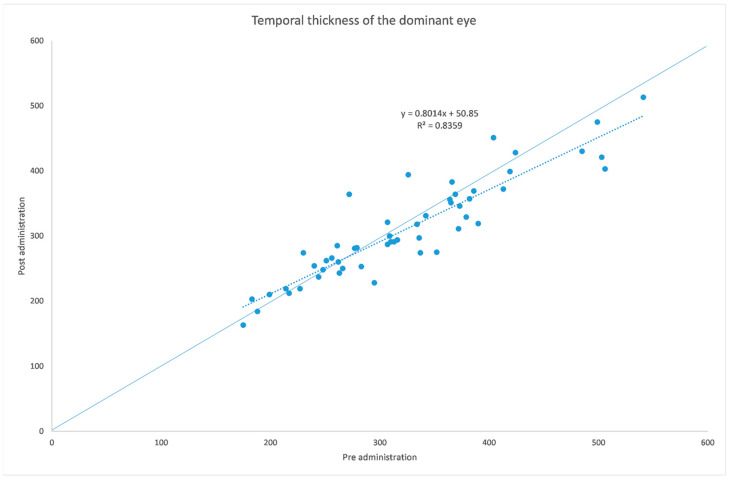
Choroidal thickness at temporal level before and after administration of tropicamide + phenylephrine in the treated eye.

**Figure 4:**
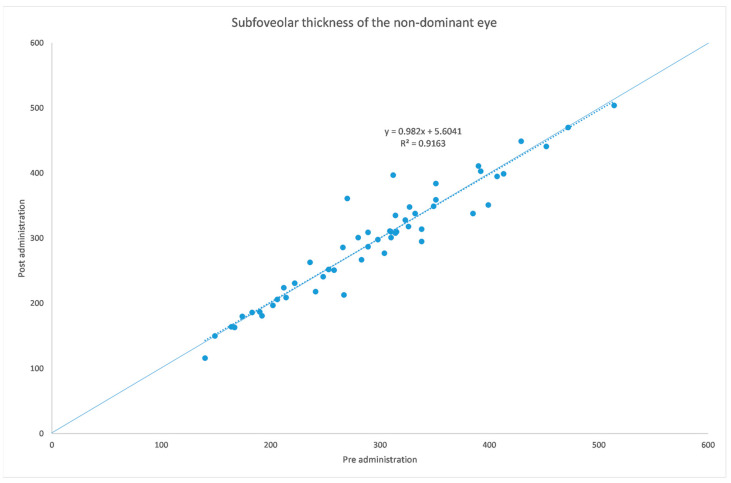
Subfoveal choroidal thickness before and after administration of tropicamide + phenylephrine in the untreated eye.

**Figure 5:**
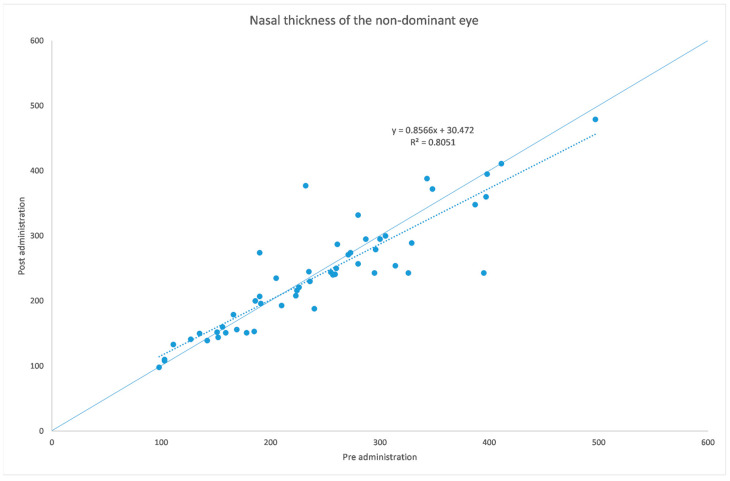
Nasal choroidal thickness before and after administration of tropicamide + phenylephrine in the untreated eye.

**Figure 6:**
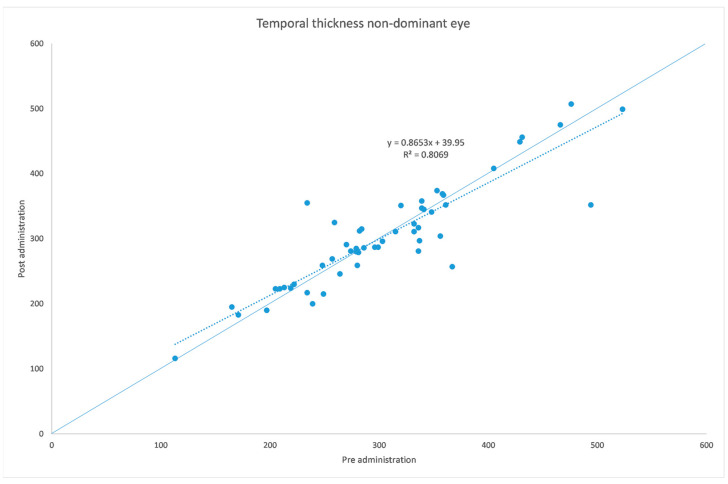
Temporal choroidal thickness before and after administration of tropicamide + phenylephrine in the untreated eye.

Table 1 and Table 2 summarize both eyes’ data before and after the administration of the mydriatic eye drops in the dominant eye. Figure 1, Figure 2, Figure 3, Figure 4, Figure 5 and Figure 6 show scatter plots showing the distribution of the data collected in both the dominant and fellow eyes at subfoveal, nasal and temporal locations.

**Figure 7:**
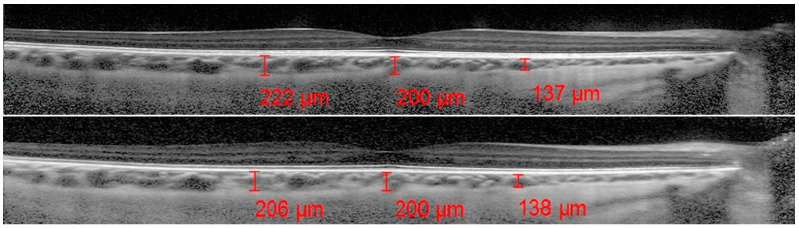
Subfoveal, nasal and temporal choroidal thickness before and after administration of tropicamide + phenylephrine.

The analyzed data, illustrated in Figure 3 and Figure 7, showed a statistically significant (*p* = 0.009) ChT reduction in the temporal sector after treatment with tropicamide + phenylephrine. In the subfoveal and nasal sectors, there were no statistically significant (*p* = 0.94; *p* = 0.85) ChT changes following the tropicamide + phenylephrine administration. A high correlation was detected between the subfoveal ChT, nasal ChT and temporal ChT in the dominant eyes before and after the tropicamide + phenylephrine administration (r = 0.921, r = 0.885, r = 0.914, all *p* < 0.001). No significant ChT changes at the subfoveal, nasal and temporal sectors were detected in the untreated eye before and after drops were administered to the fellow eye (*p* = 0.94; *p* = 0.41; *p* = 0.83). A high correlation was detected between the subfoveal ChT, nasal ChT and temporal ChT in the nondominant eyes before and after the tropicamide + phenylephrine administration in the fellow eyes (r = 0.957, r = 0.897, r = 0.898, all *p* < 0.001).

## 4. Discussion

Tropicamide + phenylephrine is a widely used mydriatic for ocular fundus visualization.

EDI OCT is the most widely used method for evaluating the choroid [14,34].

The evaluation of the change induced on the ChT by mydriatic instillation has been investigated by several studies, although with conflicting results [8,9,10,11,12,13].

Given the different results provided by the previous studies, the aim of our paper was to check the choroidal behavior following mydriatic administration. In particular, we also analyzed the fellow eye to strengthen our findings. In addition, we proposed an objective method to choose which eye should be analyzed with mydriatic eye drops through dominance tests.

The autonomic innervation of the choroid is mediated both by sympathetic and parasympathetic pathways. Vascular endothelial cells contain α adrenergic receptors, and there is also evidence of a parasympathetic innervation to choroidal nonvascular smooth muscle cells. For this reason, the administration of both phenylephrine and tropicamide, which act, respectively, as a sympathetic agonist and as an anticholinergic agent, exerting vasoconstriction on the choroidal vessels, could explain the presence of the ChT variation after their instillation [12].

The study by Casado et al. [8] on 119 eyes of 62 patients aged 59.8 ± 15.3 found that the administration of 2.5% phenylephrine caused significant thinning of the ChT in temporal and subfoveal sections, but not in nasal sections assessed by EDI OCT at time 0 and 30 min. They performed various ChT measurements at 500, 1000 and 1500 microns from the fovea.

The study by Sander et al. [9] was carried out on 14 patients aged 27.9 ± 4 with phenylephrine, homatropine and artificial tears on the right eye only. OCT scans were performed at time 0, 30 and 60 min measuring 500, 1000 and 1500 microns from the fovea. The results found a slight decrease in the ChT that was not statistically significant.

The study by Kara et al. [10] involved a sample of 90 patients, 30 of whom were given tropicamide, 30 of whom were given 2.5% phenylephrine and 30 of whom were given artificial tears three times every 5 min on a single eye. An OCT scan in EDI mode was performed after 45 min, which showed a significant reduction in the subfoveal ChT in the patients treated with tropicamide and phenylephrine.

Yuvacı et al. [11] enrolled 120 subjects and divided them into four groups, each of whom received a drop of 1% tropicamide, 2.5% phenylephrine, 1% cyclopentolate and artificial tears three times in 5 min intervals. They found a significant decrease in the ChT in the subjects receiving mydriatics, with no significant difference detected between the tropicamide, phenylephrine and cyclopentolate groups in terms of choroidal thinning.

Iovino et al. [12] recently performed a study on 95 patients with 31 receiving tropicamide, 30 receiving tropicamide + 10% phenylephrine and 34 receiving artificial tears. The authors assessed choroidal vascularity by measuring the choroidal vascularity index (CVI) and also evaluated the subfoveal thickness. The results revealed no change in vascularization.

Kim et al. [13] tested 29 individuals who were given one drop of mydriatic eye drops (5 mg/mL of tropicamide and 5 mg/mL of phenylephrine) in one eye and one drop of unpreserved saline in the contralateral eye three times in 5 min intervals, measuring the ChT at the subfoveal region and at a 0.5 mm distance from the fovea at nasal, temporal, superior and inferior locations in OCT images obtained before and after the eye drop instillation in both eyes. They found no significant changes in the ChT in either the mydriatics or placebo group in all the choroidal sections measured.

Several studies disagree with this work. The differences could be attributed to several factors, including the number of subjects examined. In the work by Sander et al. [9], 14 subjects were examined (9 males and 5 females, with a mean age of 27.9 ± 4 years), while in the work by Kim et al. [13], 29 subjects were analyzed (mean age of 33.24 ± 14.25 years). In both cases, the number of subjects involved in the studies is small.

Another factor to consider is the number of examined eyes: in the work of Casado et al. [8], the study shows that 62 patients were enrolled, but 119 eyes were examined, meaning that in some subjects, the measurements were carried out on only one eye rather than both.

The divergence in the results obtained may also be related to the dosage of the mydriatic eye drops used, as in the studies by Kara et al. [10] and Yuvacı et al. [11]. The 90 participants involved in Kara et al.’s study [10] were divided into three groups, receiving one drop of 1% tropicamide, 2.5% phenylephrine and artificial tears. Similarly, in Yuvacı et al.’s work [11], the 120 individuals were divided into four groups, treated with one drop of 1% tropicamide, 2.5% phenylephrine, 1% cyclopentolate and artificial tears. In both studies, different concentrations of mydriatic drops were used compared to our study (0.5% tropicamide + 10% phenylephrine).

Finally, a reason for the divergence in the results obtained could be attributed to the points of measurement of the ChT. In the studies by Kara et al. [10] and Iovino et al. [12], measurements of the ChT were made exclusively at the subfoveal level, and both the nasal and temporal sectors were not assessed. In addition, Iovino et al. [12] performed the measurements on the volume OCT scans, which consist of the high-speed but lower-resolution scanning of successive sections of tissue in a 30° × 20° area from the center of the fovea, whereas in our study, the measurements were performed on section OCT scans, which consist of a single scan at the foveolar site with a higher resolution that allows for greater precision in measuring the ChT.

Our results agree with the studies of Kara et al. [10], Yuvacı et al. [11] and Iovino et al. [12], whereas Casado et al. [8]. and Kim et al. [13]. did not show a statistically significant reduction in the ChT. We believe the small sample evaluated in the papers by Casado and Kim could affect their results.

A limitation of this study could be represented by the influence of the AL on the ChT. In fact, in high myopic eyes, which have a thinner ChT, the eventual choroidal thinning could be not as significant as that occurring in emmetropic eyes. This could also explain why in a thicker choroid the change seems to be higher, as shown in Figure 1, Figure 2, Figure 3, Figure 4, Figure 5 and Figure 6. Further studies providing an analysis of the samples stratified according to the AL will be necessary to investigate the ChT behavior in long eyes. Another limitation of this paper was represented by the ChT measurement only in the subfoveal, nasal and temporal regions; however, further studies also considering the superior and inferior sectors could be useful to better understand ChT behavior [35].

## 5. Conclusions

In conclusion, this study found a significant thinning of the ChT in the temporal sector following the administration of tropicamide + phenylephrine.

There are several hypotheses that could justify our results:

(1) The presence of the optic nerve in the nasal choroidal side could influence the ChT, causing a failed ChT reduction due to a “clutter effect”;
(2) An asymmetric choroidal watershed system between the temporal choroid and other areas could cause a different response to mydriatic drops [35];
(3) An asymmetrical distribution of choroidal vessels with thicker vessels on the temporal side could justify the major thinning of the temporal ChT [35].

This is a very important finding because, in the case of studies on the ChT, a choroidal assessment would be preferable before administering the tropicamide + phenylephrine eye drops.

Another important finding is that the reduction at the subfoveal level is not statistically significant (*p* > 0.05) as this result should not compromise the AL evaluation; in fact, any significant reduction in the ChT at the foveal level would result in the possibility of errors in measuring the AL, given the risk of overestimating it. This is a very important finding because it is well known that not only keratometry but also AL reliability [36,37] is fundamental to obtain an accurate IOL power calculation [37,38]. If the lens opacites are so dense that they hinder the AL measurement by optical biometry, immersion ultrasound biometry can also be carried out following the administration of tropicamide + phenylephrine eye drops. In fact, these would not induce significant ChT changes at the foveal area.

## Figures and Tables

**Table 1:** Choroidal thickness characteristics of the dominant eye.

		Preadministration (Micron)	Postadministration (Micron)	*p* Value
**Fovea**	Mean SD Median Range	303.49 81.24 298.00 151.00–489.00	303.17 80.46 300.00 143.00–478.00	0.94
**Nasal** **sector**	Mean SD Median Range	254.70 84.52 247.00 97.00–480.00	253.70 72.53 258.00 100.00–420.00	0.85
**Temporal sector**	Mean SD Median Range	323.75 88.01 313.00 175.00–541.00	310.32 77.15 294.00 163.00–513.00	0.009

The mean, standard deviation (SD), median and range of ChT were assessed at the level of the fovea and 1500 microns nasally and temporally from the fovea in the dominant drug-treated eye.

**Table 2:** Choroidal thickness characteristics of the nondominant eye.

		Preadministration (Micron)	Postadministration (Micron)	*p* Value
**Fovea**	Mean SD Median Range	295.64 85.75 304.00 140.00–514.00	295.92 87.97 301.00 116.00–504.00	0.94
**Nasal** **sector**	Mean SD Median Range	244.28 90.24 236.00 98.00–497.00	239.72 86.15 241.00 98.00–479.00	0.41
**Temporal sector**	Mean SD Median Range	304.98 84.20 296.00 113.00–523.00	303.85 81.11 296.00 116.00–507.00	0.83

The mean, standard deviation (SD), median and range of ChT were assessed at the level of the fovea and 1500 microns nasally and temporally from the fovea in the nondominant non-drug-treated eye.

## Data Availability

The datasets generated and analyzed during the current study are available from the corresponding author upon reasonable request.
